# Job crafting, work engagement, and psychological distress among Japanese employees: a cross-sectional study

**DOI:** 10.1186/s13030-017-0091-y

**Published:** 2017-02-10

**Authors:** Asuka Sakuraya, Akihito Shimazu, Hisashi Eguchi, Kimika Kamiyama, Yujiro Hara, Katsuyuki Namba, Norito Kawakami

**Affiliations:** 1 Department of Mental Health, Graduate School of Medicine, The University of Tokyo, 7-3-1, Hongo, Bunkyo-ku, Tokyo 113-0033 Japan; 2 JUKI Corporation, Tokyo, Japan

**Keywords:** Employee well-being, Job crafting, Job demands-resources model, Psychological distress, Work engagement

## Abstract

**Background:**

Job crafting, an employee-initiated job design/redesign, has become important for employees’ well-being. However, most studies on the relationship between job crafting and employees’ well-being have been conducted in western countries; thus, it is unclear whether job crafting can be effectively applied to Asian cultures, such as Japan, which emphasizes group harmony. The aim of this study was to examine the cross-sectional associations of self-reported job crafting with work engagement and psychological distress among employees in Japan.

**Method:**

A questionnaire survey through the internet was conducted among all employees of a manufacturing company in Japan. We analyzed the data from 894 respondents, all employees with regular employment. Job crafting, work engagement, and psychological distress were assessed using the Japanese version of the Job Crafting Questionnaire, the Japanese version of the Utrecht Work Engagement Scale (UWES), and the Brief Job Stress Questionnaire (BJSQ), respectively.

**Result:**

Hierarchical multiple regression showed that increasing structural job resources, social job resources, and challenging job demands was significantly and positively associated with work engagement (*β* = 0.31, *p* < 0.001; *β* = 0.14, *p* < 0.001; *β* = 0.36, *p* < 0.001, respectively). For psychological distress, increasing structural job resources was significantly and negatively associated with psychological distress (*β* = -0.15, *p* < 0.001).

**Conclusion:**

Our study suggests that increasing structural job resources is associated with higher work engagement and lower psychological distress. In addition, increasing social job resources and challenging job demands are also associated with higher work engagement.

## Background

In recent years, working conditions have been changing, particularly due to a global shift from a manufacturing economy to a service and knowledge economy with advanced information technology. With these changes, employees are more likely to feel pressure and stress at work [1]. In addition, a manager would no longer be able to design a job for employees considering their needs and skills [1]. Accordingly, an important role of employees is to design their own job or work environment [1–3]. Japanese companies have also adopted this new trend, as many companies in Japan expect their employees to have more responsibility and autonomy for improving their own employability [4, 5].

The field of occupational health has started to pay increased attention to the concept of job crafting, i.e., an idea of employee-initiated job design/redesign. Wrezesniewski and Dutton [6] have defined job crafting as “the physical and cognitive change individuals make in the task or relational boundaries of their work”, which involves changing work task, changing interpersonal relationship at work, and changing cognitions about work. By using job-crafting behaviors, employees design and improve their work and social environment in workplace by themselves [6, 7].

A further classification of job crafting has been made based on dimensions of work and work environment on which job crafting focuses, such as types of job demands and job resources based on the Job Demands-Resources (JD-R) model [7–9]. Job demands refer to physical, psychological, social, or organizational aspects of the job that require sustained physical or psychological efforts and skills and are associated with certain physical or psychological costs [8]. Recently, Van den Broeck et al. [10] distinguished between challenging job demands and hindering job demands because they have different associations with outcomes (e.g., work engagement, burnout). Challenging job demands, such as time pressure and workload, stimulate employee motivation because employees feel satisfaction from accomplishing challenging tasks, while hindering job demands, such as resource inadequacy, role ambiguity, and interpersonal conflict, have a negative effect on the mental or physical health employees [10, 11]. Job resources are physical, social, or organizational aspects of the job that help individuals achieve their working goals, stimulate personal growth, learning, and development, or reduce job demands or associated physical or psychological costs [8]. Using this refined classification of job demands and resources at work, Tims et al. [7] empirically categorized job crafting into the following four factors: (1) increasing structural job resources (e.g., autonomy, variety, and opportunity for development), (2) increasing social job resources (e.g., social support, supervisory coaching and feedback), (3) increasing challenging job demands (e.g., new project, learning new things), and (4) decreasing hindering job demands (e.g., fewer cognitive demands or stressful relationships).

Considering the categorization of job crafting proposed by Tims et al. [7, 12], both increasing structural job resources and increasing social job resources are behaviors and actions aimed at increasing job resources, which could enhance employee motivation and energy at work. Employees with high job resources tend to experience higher levels of work engagement (i.e., an active, positive, work-related state characterized by vigor, dedication, and absorption) [13]. Indeed, a positive association of increasing structural and social job resources with work engagement has been observed in previous studies in Europe [7, 12]. Hence, we proposed the following two hypotheses:

*Hypothesis 1a: Increasing structural job resources would be positively related to work engagement.*

*Hypothesis 1b: Increasing social job resources would be positively related to work engagement.*

Among job crafting behaviors targeting on job demands, increasing challenging job demands could motivate employees to develop their skill and knowledge and to achieve more challenging goals [7]. Challenging job demands was positively associated with work engagement in a previous study [14]. Hence, we proposed:

*Hypothesis 1c: Increasing challenging job demands would be positively related to work engagement.*

In contrast, by decreasing hindering job demands, employees could organize their work to alleviate perceived work pressures [7]. Hindering job demands is known to be negatively associated with work engagement [14]. Hence, we proposed:

*Hypothesis 1d: Decreasing hindering job demands would be positively associated with work engagement.*

On the other hand, job crafting may decrease negative aspects of mental health, such as psychological distress, as well as improve positive aspects, such as work engagement. Previous studies revealed that increasing structural and social job resources and increasing challenging job demands are negatively related to burnout and work-related boredom [7, 15]. In contrast, hindering job demands have been found to be associated with negative aspects of employees’ mental health [11]. An increase in hindering job demands, such as overload and emotional demands, was positively related to burnout after 1 year [16]. Hence, we proposed the following four hypotheses:

*Hypothesis 2a: Increasing structural job resources would be negatively related to psychological distress.*

*Hypothesis 2b: Increasing social job resources would be negatively related to psychological distress.*

*Hypothesis 2c: Increasing challenging job demands would be negatively related to psychological distress.*

*Hypothesis 2d: Decreasing hindering job demands would be negatively related to psychological distress.*

### The aims of this study

Previous empirical studies on the relationship between job crafting and employee well-being were conducted mainly in the Netherlands [7, 12, 15, 17], and only a few studies have been conducted in Asian countries. A study carried out in Taiwan [18] reported a positive association between job crafting and work engagement among hotel employees. However, the workplace social structure in Asian countries is more vertical [19] and hierarchy-oriented [20]. Keeping harmony in a group is an important task in the Asian cultures [21]. Thus, it is not clear whether job crafting, which is an individual effort to alter his/her design of the job and social environment in the workplace, can be applied equally effectively in Asian cultures as in western countries. More studies are needed to examine whether the concept of job crafting can be cross-culturally applicable to Asian countries like Japan. The aim of the present cross-section study was to investigate the relationship between job crafting and positive (i.e., work engagement) and negative (i.e., psychological distress) aspects of mental health among employees in Japan, focusing on the above-mentioned hypotheses.

## Methods

### Study design

The present study was a cross-sectional design. The ethics review board of The University of Tokyo approved the procedures before the start of the study. The present study conformed to the STROBE checklist.

### Participants

A questionnaire survey through the Internet was conducted among all employees of a manufacturing company in Japan during a health examination in November 2013. Overall, 972 employees completed the survey. The overall response rate was 99.9%. We excluded 78 respondents (8% of the total respondents) because of non-regular employment or reemployment in a temporary position and analyzed the data from 894 respondents. Before starting the study, we explained the content of the study to all participants through supervisors and a bulletin board, after which they gave informed consents in an opt out form. Their anonymity was preserved.

### Measures

#### Job crafting

Job crafting was assessed using the Japanese version of the job crafting questionnaire, which was reported as reliable and valid [22]. It comprises four subscales, increasing structural job resources (five items, e.g., ‘I try to learn new things at work.’), increasing social job resources (five items, e.g., ‘I ask colleagues for advice.’), increasing challenging job demands (five items, e.g., ‘When there is not much to do at work, I see it as a chance to start new projects.’), and decreasing hindering job demands (six items, e.g., ‘I organize my work in such a way to make sure that I do not have to concentrate for too long a period at once.’). All items were scored on a five-point Likert scale ranging from 1 (never) to 5 (very often), and a total score for each subscale was calculated.

#### Psychological distress

Psychological distress was assessed using the Brief Job Stress Questionnaire (BJSQ) [23], which consists of 15 items, reflecting irritation (three items, e.g., ‘I feel anger.’), fatigue (three items, e.g., ‘I feel very tired.’), anxiety (three items, e.g., ’I feel uneasy.’), and depression (six items, e.g., ‘I feel depressed.’). All items were scored on a four-point Likert scale ranging from 1 (never) to 4 (almost always), and the total score was calculated.

#### Work engagement

Work engagement was assessed using the Japanese version of the Utrecht Work Engagement Scale (UWES), which was reported as reliable and valid [24]. It consists of nine items, reflecting vigor (three items, e.g., ‘At my work, I feel bursting with energy.’), dedication (three items, e.g., ‘My job inspires me.’), and absorption (three items, e.g., ‘I get carried away when I am working.’). All items were scored on a seven-point Likert scale ranging from 0 (never) to 6 (always), and the total score was calculated.

#### Other variables

Other covariates at baseline included age, gender, and job position. Age was categorized as under 20 years, 20–29 years, 30–39 years, 40–49 years, and over 50 years. Gender was classified into male or female. Job position was classified as non-manager, lower-level manager (i.e., managers having no subordinates), middle-level manager (i.e., section chief), and top management (i.e., those above the head of department).

### Sample size calculation

Post-hoc sample size calculation was conducted in the present study. There is no appropriate previous study that can be used to estimate correlations of job crafting factors (increasing structural job resources, increasing social job resources, increasing challenging job demands, decreasing hindering job demands) with work engagement. Thus, with a population of 894, we have 98.8% power to detect a predictive value, assuming that *α* error = 0.05, effect size *f* ^*2*^ = 0.02.

### Statistical analysis

We analyzed the data from 894 respondents after excluding those with non-regular employment and re-employment in a temporary position. The Internet survey system did not allow missing values; therefore, respondents had to answer all of the questions. A hierarchical multiple regression analysis, which can show the associations between an independent and a dependent variable, controlled for all other predictors included in the analysis, was carried out on work engagement and psychological distress. The independent variables were entered into the equation in two steps. Demographic characteristics (age, gender, job position) were entered in Step 1 and the four factors of job crafting were entered in Step 2 simultaneously. Statistical analyses were conducted by SPSS 22.0J for Windows.

## Result

### Characteristics of respondents

Table 1 shows the respondents’ characteristics. Most were male (84%), older than 40 years of age (67%), and non-managers (77%).

**Table 1:** Sociodemographic characteristics of the respondents in the study (Total *N* = 894)

	Number	Percent
1 Gender		
Male	751	(84.0)
Female	143	(16.0)
2 Age(years)		
Under 20 years	2	(0.2)
20–29	119	(13.3)
30–39	174	(19.5)
40–49	323	(36.1)
Over 50 years	276	(30.9)
3 Job position		
Non-manager	688	(77.0)
Lower-level manager	67	(7.5)
Middle-level manager	110	(12.3)
Top management	29	(3.2)

### Correlations of the variables and Cronbach’s alpha coefficient

Table 2 shows means, standard deviations, Cronbach’s alpha coefficients, and correlations among the variables used in the study. Increasing structural job resources, increasing social job resources, and increasing challenging job demands correlated positively with work engagement. Increasing structural job resources was negatively associated with psychological distress, whereas decreasing hindering job demands was positively associated with psychological distress. Cronbach’s alpha coefficients of each measure were all above 0.70.

**Table 2:** Mean, SD, internal consistencies (Cronbach’s alpha), and correlations of the variables (*N* = 894)^a)^

	Measures	Range	Mean	SD	Cronbach’s alpha	1	2	3	4	5	6	7	8	9
1	Age^b)^						−0.09**	0.38***	−0.08*	−0.25***	0.07*	−0.11**	0.12***	−0.03
2	Gender^c)^							−0.20***	−0.12***	−0.01	−0.13***	−0.06	0.03	0.00
3	Job position^d)^								0.10**	0.00	0.23***	−0.11***	0.12***	0.04
4	Increasing structural job resources	5–25	14.2	4.2	0.90					0.37***	0.61***	0.22***	0.50***	−0.11**
5	Increasing social job resources	5–25	9.1	3.0	0.76						0.42***	0.32***	0.30***	0.01
6	Increasing challenging job demands	5–25	10.6	3.9	0.84							0.25***	0.54***	−0.05
7	Decreasing hindering job demands	5–30	12.4	4.1	0.80								0.00	0.18***
8	Work engagement	0–54	23.4	9.2	0.93									−0.31***
9	Psychological distress	1–60	29.9	8.5	0.93									

***p < 0.001 **p < 0.01 *p < 0.05

^a)^Responses for the items were summed up for increasing structural job resources, increasing social job resources, increasing challenging job demands, decreasing hindering job demands, work engagement, and psychological distress, respectively

^b)^1 = Under 20 years, 2 = 20–29 years, 3 = 30–39 years, 4 = 40–49 years, 5 = over 50 years

^c)^1 = Male, 2 = Female

^d)^1 = Non-manager, 2 = Lower-level manager, 3 = Middle-level manager, 4 = Top management

### Hierarchical multiple regression analyses

Table 3 summarizes the results of hierarchical multiple regression analyses predicting work engagement and psychological distress from demographics (age, gender, and job position) and job crafting factors. For work engagement, explained variances (*R* ^*2*^) changed significantly from Step 1 to Step 2. In Step 2, increasing structural job resources, increasing social job resources, and increasing challenging job demands were positively associated with work engagement, while decreasing hindering job demands was negatively associated with work engagement. For psychological distress, the change in explained variances (*R* ^*2*^) from Step 1 to Step 2 was also significant. In Step 2, increasing structural job resources was negatively associated with psychological distress while decreasing hindering job demands was positively associated with psychological distress. The other two job crafting factors (i.e., increasing social job resources and increasing challenging job demands) were not significantly associated with psychological distress.

**Table 3:** Hierarchical multiple regression analyses predicting work engagement and psychological distress from demographics, job crafting. (*N* = 894)^a^

Predictors	Work engagement		Psychological distress	
	Steps		Steps	
	I	II	I	II
Age^b^	0.09**	0.17***	−0.06	−0.06
Gender^c^	0.06	0.11***	0.00	0.00
Job position^d^	0.09*	−0.06*	0.06	0.11**
Increasing structural job resources		0.31***		−0.15***
Increasing social job resources		0.14***		−0.01
Increasing challenging job demands		0.36***		−0.03
Decreasing hindering job demands		−0.19***		0.23***
*R* ^2^	0.02***	0.40***	0.00	0.06***
Change in *R* ^2^		0.38***		0.06***

***p < 0.001 **p < 0.01 *p < 0.05

^a^The *β* values are the standardized coefficients

^b^1 = Under 20 years, 2 = 20–29 years, 3 = 30–39 years, 4 = 40–49 years, 5 = over 50 years

^C^1 = Male, 2 = Female

^d^1 = Non-manager, 2 = Lower-level manager, 3 = Middle-level manager, 4 = Top management

## Discussion

The present study examined the relationship of four different types of job crafting with positive mental health (i.e., work engagement) and negative mental health (i.e., psychological distress) among Japanese employees. The results of the hierarchical multiple regression analyses showed positive associations of increasing structural job resources, social job resources, and challenging job demands with work engagement and a negative association of increasing structural job resources with psychological distress. Unexpectedly, decreasing hindering job demands was negatively associated with work engagement and positively with psychological distress.

Our findings were line with Hypothesis 1a, 1b, and 1c, which proposed that increasing structural job resources, social job resources, and challenging job demands are positively related to work engagement (Hypothsis1a, 1b, and 1c respectively). These results are consistent with previous studies of Tims et al. [7, 12]. Furthermore, they indirectly support the concept of the JD-R model through positive associations of job resources and challenging job demands with work engagement [8, 14, 25].

However, our results were not in line with Hypothesis 1d, decreasing hindering job demands was negatively associated with work engagement (Table 3), although the bivariate correlation between them was not significant (Table 2). Several reasons can be considered to explain this unexpected result. First, multi-collinearity could have contributed to this result. However, the correlations between decreasing hindering job demands and other job crafting variables were low (Table 2), and the variance inflation factor (VIF) predicting work engagement from decreasing hindering job demands in hierarchical multiple regression analysis was also low (VIF = 1.17). Therefore, multi-collinearity was not likely to affect our result. A second explanation may be reversed causation due to our cross-sectional study design. Employees with low work engagement may have been exposed to high hindering job demands, thus trying to decrease hindering job demands. Further longitudinal study is needed to examine the effect of decreasing hindering job demands at baseline on work engagement at follow-up, which could clarify the causal relationship between them. Finally, we can speculate a non-linear association of decreasing hindering job demands with work engagement. To explore the curvilinear relationship between decreasing hindering job demands and work engagement, we conducted multiple regression analysis in which we entered the decreasing hindering job demands as a simple (in order to control for the linear effect) and squared scores of decreasing hindering job demands. The analyses showed that the standardized beta of squared decreasing hindering job demands (*β* = −0.43; *p* < 0.01) was significant. A linear effect remained positive and significant after entering the squared score of decreasing hindering job demands in multiple regression predicting work engagement (*β* = 0.41; *p* < 0.01). The negative sign of the regression weight for the squared parameter implies that a moderate level of decreasing hindering job demands was associated with the highest level of work engagement, whereas very high and very low decreasing hindering job demands were associated with lower levels of work engagement. Future study needs to examine this curvilinear relationship in more detail.

Regarding the relationship between job crafting and negative mental health (i.e., psychological distress), increasing structural job resources was negatively associated with psychological distress, in line with Hypothesis 2a. Whereas previous studies clarified the effects of increasing structural job resources on work-related outcomes, such as burnout and work-related boredom [12, 15], our results consider the effect on more general outcomes like psychological distress.

However, not in line with Hypothesis 2b and 2c, increasing social job resources and challenging job demands were not significantly associated with psychological distress. This result contradicts a previous study [12], which showed that these two types of job crafting contributed to lower burnout. Although we do not know the reasons for this inconsistent result, we can at least say that these two types of job crafting may not lead to lower psychological distress. Apart from coping, the primary focus of which is to manage a stressful situation and/or its consequent distress via cognitive and behavioral efforts [26], job crafting focuses mainly on improving the meaning of one’s own job rather than dealing with stress. Future study needs to examine the role of job crafting in dealing with stress in more detail.

In addition, our findings were not in line with Hypothesis 2d, as we found that decreasing hindering job demands was positively associated with psychological distress. Similar explanations may be offered for this unexpected result. First, multi-collinearity could have contributed to this result. However, the correlations between decreasing hindering job demands and other job crafting variables were low (Table 2). Furthermore, the VIF of the regression predicting psychological distress from decreasing hindering job demands was also low (VIF = 1.17). A second explanation may be the reversed causation. Employees with high psychological distress may have already experienced high job demands; thus, they may have tried to decrease hindering job demands. Hence, further longitudinal study to investigate the effect of decreasing hindering job demands at baseline on psychological distress at follow-up is needed. Finally, the association of decreasing hindering job demands with psychological distress could have been non-linear. To explore the curvilinear relationship between decreasing hindering job demands and psychological distress, we again conducted multiple regression analysis in which we entered the decreasing hindering job demands as simple (in order to control for the linear effect) and squared scores of decreasing hindering job demands. The analyses showed that the standardized beta of squared decreasing hindering job demands (*β* = −0.47, *p* < 0.01) was significant. A linear effect remained positive and significant after entering the squared score of decreasing hindering job demands in multiple regression predicting psychological distress (*β* = 0.64, *p* < 0.001). The negative sign of the regression weight for the squared parameter implies that a moderate level of decreasing hindering job demands was associated with the highest level of psychological distress, whereas very high and very low decreasing hindering job demands were associated with lower levels of psychological distress. Again, future study needs to examine this curvilinear relationship in more detail.

As for practical implications, given our findings, a job-crafting training program aimed at increasing job resources would be a promising way to improve work engagement and also burnout (i.e., job-related distress), which are assumed to be opposite aspects of work engagement.

## Limitations and future directions

Our study has several limitations. First, because we used a self-report questionnaire, common method bias could have affected the results. For example, participants may have answered the questionnaire in a socially desirable manner, which could have led to the overestimation of the true associations. It would be useful to add other measurements, such as peer-rating or supervisor-rating behavior, to future studies. However, Spector [27] stated that social desirability is unlikely to significantly inflate the relationships. Thus, the effect of social desirability on our results may be small, although we cannot rule it out completely.

Second, we could not control for other unmeasured variables. For example, proactive personality, a tendency to behave proactively in various situations, has been reported to be related to job crafting [28]. In addition, Demerouti [2] stated that job crafting occurs among employees who are proactive, motivated by growth, or experience misfit between their motivational style and the environmental cues. Thus, in further study, we should consider these individual factors.

Third, due to its cross-sectional design, we could not determine the causality between job crafting and outcome variables. For example, although we showed a positive relationship between job crafting (increasing structural job resources, social job resources, challenging job demands) and work engagement, it is unclear whether highly engaged employees would be apt to engage in job crafting or whether job crafting may increase work engagement. Therefore, a longitudinal study is needed to determine if job crafting at baseline affect work engagement and other outcome variables at follow-up.

Fourth, our participants were all employees of a single Japanese manufacturing company that agreed to cooperate on this survey, which limits the generalization of our results. Future study needs to examine whether our findings can be applicable to other occupations and/or workers in other countries, especially in terms of the curvilinear relationship between decreasing hindering job demands and outcomes (e.g., work engagement and psychological distress).

Finally, the current regression analysis only examines the direct association of job crafting with work engagement and psychological distress. In further study, mediator analyses could be applied in order to clarify the mechanism behind the relation. Moderator analyses are also needed to clarify individual differences in the relationship and under which situations job crafting can be more effective.

## Conclusion

Our findings indicate that increasing structural and social job resources and increasing challenging job demands is associated with higher work engagement among Japanese employees. In addition, increasing structural job resources is also associated with lower psychological distress. Future study is needed to investigate the effect of job crafting on the positive and negative aspects of employee mental health.
